# Patient-reported outcomes on familial amyloid polyneuropathy (FAP)

**DOI:** 10.1186/s13023-020-01575-6

**Published:** 2020-10-14

**Authors:** Fabian J. Bolte, Christel Langenstroer, Frauke Friebel, Anna Hüsing-Kabar, Martin Dugas, Hartmut H. Schmidt

**Affiliations:** 1 Medizinische Klinik B (Gastroenterologie, Hepatologie, Endokrinologie, Klinische Infektiologie), Universitätsklinikum Münster, Albert-Schweitzer-Campus 1, Gebäude A14, 48149 Münster, Germany; 2 Institut für Medizinische Informatik, Universitätsklinikum Münster, Münster, Germany

**Keywords:** Transthyretin, Amyloidosis, Patient reported outcomes, Disease burden, Disability

## Abstract

**Background:**

Transthyretin familial amyloid polyneuropathy (ATTR-FAP) is a rare autosomal dominant inherited disease affecting multiple organ systems. ATTR-FAP patients’ experiences have rarely been documented. The aim of this study was to collect patient reported outcomes across different countries to assess unmet needs and challenges. An anonymous survey was conducted at the 2nd European meeting on ATTR amyloidosis in Berlin in September 2019. Survey questions captured information on demographics, clinical characteristics, diagnostic experience, quality of life, disability and ATTR-FAP management.

**Results:**

A total of 38 ATTR-FAP patients from 15 different countries participated in the survey. ATTR-FAP had a substantial impact on patients’ day-to-day life, including difficulties in standing, walking, and participation in community activities. It also had negative effects on the mental health of patients. The survey highlighted several unmet needs and challenges from a patients’ perspective, including (i) a need for increased awareness and a standardized diagnostic pathway, (ii) a need for better treatment access and supportive care and (iii) a need for better information about research and clinical trials.

**Conclusions:**

This global patient survey provides valuable findings to address ATTR-FAP patients’ needs and challenges in order to further the goal of patient-centered care.

## Background

Transthyretin familial amyloid polyneuropathy (ATTR-FAP) is an autosomal dominant inherited condition with over 100 different mutations identified worldwide in the transthyretin (TTR) gene [1, 2]. The most common mutation is Val30Met [1]. ATTR-FAP is estimated to affect 10,186 people worldwide. The prevalence varies across geographic regions and is higher in endemic countries like Portugal, Japan, Sweden and Brazil [3–5].

ATTR-FAP is characterized by the deposition of TTR amyloid fibrils in different organ systems [1, 5]. The most common form of ATTR-FAP affects the peripheral and autonomic nervous system, resulting in a length-dependent peripheral neuropathy. Thus, ATTR-FAP patients can present with various symptoms including loss of sensation in the extremities, carpal tunnel syndrome, erectile dysfunction, diarrhea, constipation and orthostatic hypotension [6–8]. The cardiac form of ATTR-FAP results in progressive heart failure with arrhythmias [9]. Due to the non-specific nature of symptoms, ATTR-FAP patients may often be seen by multiple specialists, leading to delays in an accurate diagnosis and different treatment recommendations. This has a considerable impact on patients’ lives since ATTR-FAP leads to death in approximately 10 years after the onset of symptoms without any treatment [1, 10].

Despite the considerable impact of this disease on patients' lives, the experiences and challenges of ATTR-FAP patients have rarely been documented. However, patient reported outcomes are critical for the adequate management of patients because physicians tend to underestimate symptom and disease severity by clinical evaluation [11]. This may lead to inaccurate assessments and treatment decisions resulting in poor health-related quality of life. Thus, patient reported outcomes, in addition to the modified neuropathy impairment score, echocardiography and measurement of N-terminal pro-brain natriuretic peptide [12–15], are invaluable to set individual treatment goals and monitor the response to specific interventions in patients with ATTR-FAP.

The International Consortium for Health Outcomes Measurement (ICHOM) brings together international teams of patients, physicians and researchers to define outcomes that matter most to patients [16]. It is believed that measuring and reporting standardized patient outcomes across multiple sites will help to address insufficiencies in health care and thus improve the performance of the health care system worldwide [17].

To our knowledge, this has been the first study to collect ATTR-FAP patient reported outcomes across different countries. The aim of our survey was to study experiences, quality of life, and functional impairment of ATTR-FAP patients in order to highlight their unmet needs and challenges.

## Results

### Patient characteristics and experiences

A total of 38 patients diagnosed with ATTR-FAP who attended the 2nd European meeting on ATTR-FAP in Berlin participated in the survey (long version n = 34, short version n = 4). These patients were recruited from 15 different countries including Australia (n = 1), Austria (n = 2), Brazil (n = 1), Canada (n = 2), Denmark (n = 1), England (n = 6), France (n = 2), Germany (n = 5), Italy (n = 1), Netherlands (n = 5), New Zealand (n = 1), Portugal (n = 3), Sweden (n = 2), Switzerland (n = 1) and Venezuela (n = 1). Overall, the majority of participants in the survey were from Europe (n = 27; 79%). Patient characteristics are summarized in Table 1. The mean age of patients with ATTR-FAP was 53.3 years and 79% were men. The majority of patients were living together with a partner (79%) rather than alone (21%). More than half of the patients were currently working full-time (38%) or part-time (21%) whereas 23% of patients were unable to work due to ATTR-FAP. 18% of patients were not working by choice, most of them being retired.

**Table 1:** Summary of patient characteristics

Patient characteristics	
Age (n = 38)	Mean 53.3 years, SD +/− 12.6
Gender (n = 38)	Male 79% Female 21%
Country (n = 34)*	Australia (n = 1) Austria (n = 2) Brazil (n = 1) Canada (n = 2) Denmark (n = 1) England (n = 6) France (n = 2) Germany (n = 5) Italy (n = 1) Netherlands (n = 5) New Zealand (n = 1) Portugal (n = 3) Sweden (n = 2) Switzerland (n = 1) Venezuela (n = 1)
Working status (n = 34)*	Full-time 38.2% (n = 13) Part-time 20.6% (n = 7) Not working by choice 17.6% (n = 6) Unable to work due to ATTR-FAP 23.5% (n = 8)
Living status (n = 34)*	Alone 21% (n = 27) Partner 79% (n = 7) Nursing home 0% (n = 0)
Smoking history (n = 34)*	Current smoker 0% (n = 0) Ex-smoker 21% (n = 7) Non Smoker 79% (n = 27)
Alcohol history (n = 34)*	Consumption of alcohol drinks or beverages Never 32% (n = 11) 1 to 3 days per month 21% (n = 7) 1 to 2 days per week 26% (n = 9) 3 to 4 days per week 21% (n = 7) 5 to 6 days per week 0% (n = 0) Every day 0% (n = 0)
ATTR diagnosis (n = 32)**	Biopsy and genetic test 62.5% (n = 20) Biopsy alone 15.6% (n = 5) Genetic test alone 15.6% (n = 5) None 6.3% (n = 2)
ATTR mutation (n = 32)**	p.TTRV30M 46.9% (n = 15) Non p.TTRV30M 21.9% (n = 7) Unknown 31.2% (n = 10)
Organ systems involved (n = 32)**	Nerves 78.1% (n = 25) Heart 59.4% (n = 19) Gastrointestinal tract 37.5% (n = 12) Kidneys 18.8% (n = 6) Eyes 18.8% (n = 6)
Health care visits made before diagnosis of ATTR-FAP (n = 32)**	Median 3, IQR 1-15 (range 1–50)
Number of years before diagnosis of ATTR-FAP (n = 32)**	Median 2 years, IQR 1–4 (range 1–10)
Time since diagnosis (n = 32)**	Median 5,5 years, IQR 4–18 (range 1–31)

*ATTR-FAP*, Transthyretin familial amyloid polyneuropathy, *SD* standard deviation; IQR, interquartile range

^*^All patients who completed the long version of the questionnaire are included (n = 34)

^**^All patients who completed the long version of the questionnaire are included. Two patients were not responding to the question (n = 32)

Median patient reported time from first symptom onset to diagnosis was 2 years (IQR 1–4, range from 1 to 10); 25% of patients required more than 4 years for an ATTR-FAP diagnosis. During this time, patients required a median of 3 visits to health care professionals (IQR 1–15, range from 1 to 50), yet 25% of patients required more than 15 visits. These diagnostic delays are even more obvious when excluding patients who were diagnosed due to a positive family history and consecutive genetic testing. In a majority of patients (62.5%), a biopsy and genetic test were performed to confirm the diagnosis of ATTR-FAP; 31.25% of patients underwent either a biopsy or genetic test, 6.25% had neither a biopsy or genetic test. At the time of diagnosis, the most common organ systems being involved were the nervous (78%) and cardiovascular system (59%) followed by the gastrointestinal tract (38%), kidneys (19%) and eyes (19%).

Taken together, our results show that there is a significant delay between symptom onset and diagnosis of ATTR-FAP. The diagnosis was mostly but not consistently based on combined biopsy and genetic testing.

### Quality of life and functional impairment

**Fig. 1:**
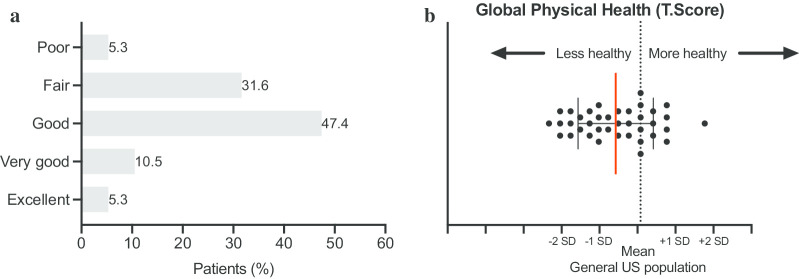
Quality of life of patients with ATTR-FAP. **a** Patient reported health score on a scale of 1 to 6 (1 = poor health, 6 = excellent health). Base population: All respondents (n = 38). Question: In general, how would you rate your health? **b** PROMIS-10. The Global Physical Health score was generated by summing responses to Global03, Global06, Global07 rescored, and Global08 rescored. Conversion tables were used to convert the Global Physical Health raw scores into T-score values on an individual respondent. Base population: All respondents (n = 38). Questions: Global02: In general, how would you rate your quality of life? Global06: To what extent are you able to carry out your everyday physical activities such as walking, climbing stairs, carrying groceries or moving a chair? Global07: In the past 7 days, how would you rate your pain on average? (Scale from 0 to 10: 0 is no pain and 10 is the worst imaginable pain) and Global08: In the past 7 days, how would you rate your fatigue on average?

**Fig. 2:**
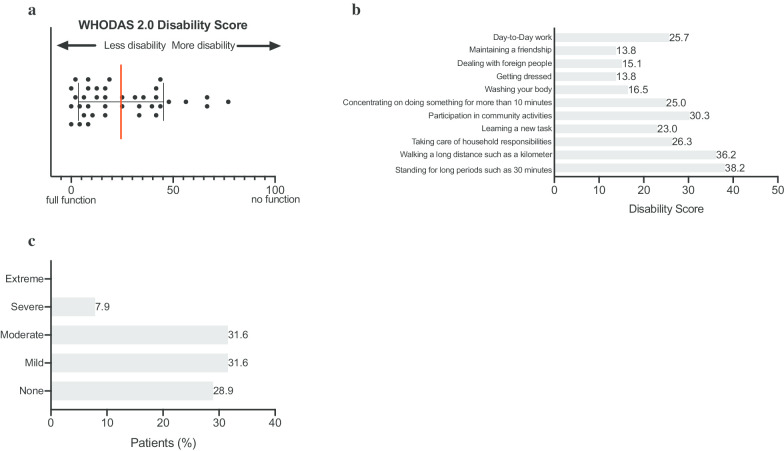
Functional impairment of patients with ATTR-FAP. **a** World Health Organization Disability Assessment Schedule 2.0 (WHODAS 2.0). The scores assigned to each of the 12 items (“none” 0, “mild” 1, “moderate” 2, “severe” 3 and “extreme” 4) are summed for each individual patient. This summed score is divided by 48 and multiplied by 100 in order to determine a functioning level ranging from 0% (full function) to 100% (no function) for each individual respondent. Base population: All respondents (n = 38). **b** World Health Organization Disability Assessment Schedule 2.0 (WHODAS 2.0). Functional impairment based on the individual items: day-to-day work, maintaining a friendship, dealing with foreign people, getting dressed, washing your body, concentrating on doing something for more than 10 min, joining in community activities, learning a new task, taking care of household responsibilities, walking a long distance such as a kilometer and standing for long periods such as 30 min. The scores assigned to each of the above-mentioned items (“none” 0, “mild” 1, “moderate” 2, “severe” 3 and “extreme” 4) are summed for each item across all patients. This summed score is divided by 152 and multiplied by 100 in order to give a disability score ranging from 0% (full function) to 100% (no function). Base population: All respondents (n = 38). **c** Patient reported mental health. Base population: All respondents (n = 38). Question: In the past 30 days, how much have you been emotionally affected by your health problems?

On a scale from 1 to 6 (1 = poor health and 6 = excellent health), ATTR-FAP patients rated their general health on average at 3, with 16% of patients reporting poor to fair health (Fig. 1a). Following conversion of the PROMIS-10 global physical health score into T-score values, the data shows that the global physical health of ATTR-FAP patients is half a standard deviation worse than the general US population. In other words, 63% of patients reported to be less healthy than the general US population (Fig. 1b). The functioning level of ATTR-FAP patients (WHODAS 2.0 Disability score: 0 = full function and 100 = no function) ranges tremendously from 0 to 77 (Fig. 2a, Median 16, IQR 8–41). A majority of patients (61%) reported difficulties with day-to-day work. As shown in Fig. 2b, the main areas of functional impairment include standing for long periods and walking a long distance. This was followed by difficulties with participation in community activities, taking care of household activities, and concentrating for longer time periods. Moreover, a large proportion of patients reported that living with ATTR-FAP affected their emotional health ranging from mild (32%), to moderate (32%), to severe (8%; Fig. 2c).

In summary, our findings show that ATTR-FAP has considerable impact on patients’ daily lives including difficulties in basic functions such as standing, walking and participation in community activities.

### ATTR-FAP Management

**Fig. 3:**
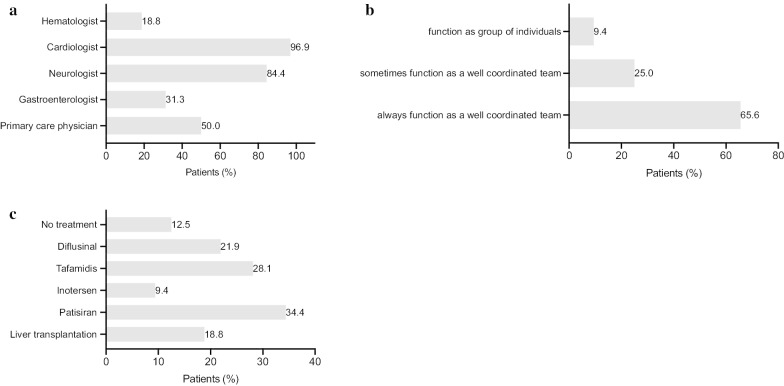
ATTR-FAP Management. **a** Health care professionals involved in the care of ATTR-FAP. Base population: Respondents of the long version of the questionnaire. Two patients were not responding to the question (n = 32). Multiple-answer question with percentages adding up to more than 100%. Question: Which of the following healthcare professionals are involved in the ongoing management of your ATTR amyloidosis? **b** Patients’ experience on the coordination of care between different specialists. Base population: Respondents of the long version of the questionnaire. Two patients were not responding to the question (n = 32). Question: Which of the following best describes the approach taken by your medical care providers in the management of your ATTR amyloidosis? **c** Treatment of ATTR-FAP patients. Base population: Respondents of the long version of the questionnaire. Two patients were not responding to the question (n = 32). Multiple-answer question with percentages adding up to more than 100%. Question: ATTR amyloidosis treatment options: Please tick all that apply

Approximately three health care professionals were involved in the ongoing management of ATTR-FAP patients, with cardiologists (96%) and neurologists (84%) seen most often (Fig. 3a). A majority of patients believed that the health care professionals involved in their management functioned as a well-coordinated team (66%). Nevertheless, there is still a substantial proportion of patients stating that the health care professionals involved in their management functioned as a group of individuals (9%) or only occasionally as a well-coordinated team (25%, Fig. 3b). Annually, patients reported having a median of 2 (IQR 2–4, range from 1–12) ATTR-FAP related health care visits.

**Fig. 4:**
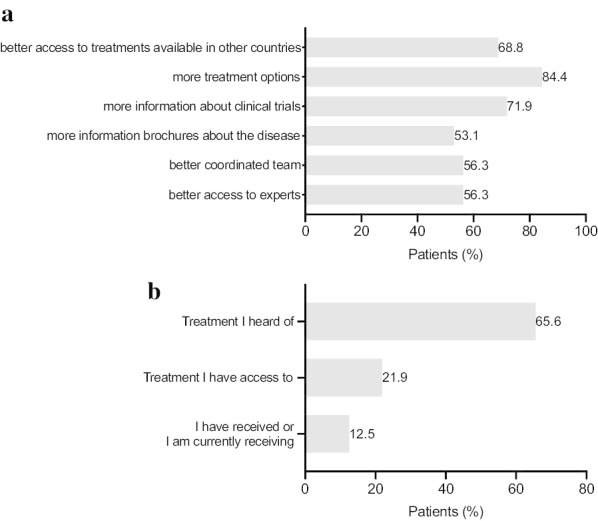
ATTR-FAP management improvements. **a** Patients’ beliefs regarding improvements that would help with the ongoing ATTR-FAP management. Base population: Respondents of the long version of the questionnaire. Two patients were not responding to the question (n = 32). Multiple-answer question with percentages adding up to more than 100%. Question: Which of the following would help with the ongoing management of your ATTR amyloidosis? **b** Physiotherapy in ATTR-FAP management. Base population: Respondents of the long version of the questionnaire. Two patients were not responding to the question (n = 32). Question: Physiotherapy in ATTR-FAP: Treatment I heard of, treatment I have access to, treatment I have received or treatment I am currently receiving. Please tick all that apply

The prospects for ATTR-FAP have changed with the approval of gene silencing drugs [18, 19]. As shown in Fig. 3c almost half of the patients participating in the survey were currently being treated with gene silencing drugs such as Patisiran (34.4%) and Inotersen (9.4%) and the other half with TTR stabilizers such as Tafamidis (28.1%) and Diflunisal (21.9%). 18.8% of patients underwent liver transplantation. It is noteworthy to mention that 25% of patients have received more than one treatment. However, access to treatment varies considerably among different countries and 12.5% of patients have not received any treatment yet. A majority of patients (68.8%) would like access to a wider range of existing ATTR-FAP treatment options (Fig. 4a). Moreover, more than half of patients (72%) desire more information about clinical trials. Keeping in mind that standing and walking difficulties are affecting patients’ daily lives, the proportion of patients who have received or are currently receiving physiotherapy was relatively small (12.5%, Fig. 4b).

In summary, our results indicate that more efforts are necessary to help secure best possible treatment and supportive care involving physiotherapy for patients with ATTR-FAP.

## Discussion

To our knowledge, this is the first survey focusing on patient reported outcomes in ATTR-FAP across different countries. This may be due to the lack of standardized and disease specific patient reported outcome measures and the relative low prevalence of the disease in countries other than Portugal, Japan, Sweden and Brazil.

This survey, including validated health-related quality of life assessment tools, such as the PROMIS-10 und WHODAS 2.0, reveals poorer health-related quality of life scores in patients with ATTR-FAP as compared to the general population [20]. ATTR-FAP has a substantial impact on patients’ day-to-day life with difficulties in standing, walking and participation in community activities. It also has a negative effect on the mental health of patients. These findings indicate that an assessment of functional impairment focusing on basic physical functions (i.e. six-minute walk test) and mental health (i.e. Patient Health Questionnaire (PHQ-9)) are essential to guide treatment decisions and to improve the quality of life in ATTR-FAP patients. Emerging therapies including gene silencing drugs and lipid nanoparticle encapsulated CRISPR/Cas9 components targeting human TTR may stabilize or even improve basic physical functions and mental health. Moreover, this survey highlights several unmet needs and challenges for patients with ATTR-FAP, including (i) a need for increased awareness and a standardized diagnostic pathway, (ii) a need for better access to the best possible treatment and supportive care including physiotherapy, ergotherapy and nutritional consulting across different countries, and (iii) a need for better information about research and clinical trials. It is noteworthy that the diagnosis of ATTR-FAP was mostly but not consistently based on combined biopsy and genetic testing. A total of 7 (22%) participants in the survey did not have a genetic test. An explanation might be that these patients had a positive family history. Nevertheless, we endorse a combined biopsy and genetic testing as an integral part of the diagnostic pathway. The Amyloidosis Alliance created in June 2018 is a network composed of 14 independent international patient organizations and groups which aims to be the global voice of patients with amyloidosis. It has formed five working groups (task forces) on awareness and diagnosis, treatment, research, communication and advocacy to address unmet needs in patients with ATTR-FAP.

This survey has several potential limitations that should be taken into consideration including (i) selection bias, given the fact that a majority of participants in the survey were from Europe (79%) and findings are solely based on patients who were able to attend the 2nd European meeting on ATTR-FAP in Berlin, (ii) recall bias, given the fact that findings are solely based on patient responses to closed-ended questions and not confirmed by medical records, and (iii) patients who participated in the survey may have been more informed and engaged with their disease. Moreover, there was a predominance of males (79%) of the respondents. Thus, the survey population may not be fully representative of a heterogenous ATTR-FAP population. However, a key strength of this survey is that findings directly reflect patients’ perspectives on ATTR-FAP.

## Conclusions

The health care system has increased efforts to provide more patient-centered care by customizing care to individual patient’s needs [21, 22]. Thus, understanding what matters most to patients is unique and of significant value. This survey provides valuable findings that may be used in future research to address ATTR-FAP patients' needs and challenges on the way to patient-centered care.

## Patients and methods

### Study design

Patients were recruited during the 2nd European meeting on ATTR-FAP for patients and doctors in Berlin from September 1st to 3rd 2019. For transparency, the survey was announced in the official program and patient meetings.

### Survey details

The domains and questions of the survey were initially generated and discussed at a local roundtable meeting of experts involved in the treatment of ATTR-FAP at the University Hospital of Münster on the basis of a literature review. We employed the Patient Reported Outcomes Measurement Information System Short Form Version 1.1 Global Health (PROMIS-10 ©2006–2017 PROMIS Health Organization) [20] to assess quality of life and the World Health Organization Disability Assessment Schedule 2.0 (WHODAS 2.0 12-item instrument) [23] to assess disability. A research collaboration was established with the Institute of Medical Informatics to generate an electronic form for the documentation of patient data and its implementation on iPad devices. The final questionnaire was reviewed and edited by all co-authors. This survey was developed to be primarily conducted on iPads with an approximate time for completion of 10 min (short version) and 20 min (long version). The survey was available in two languages: English and Spanish (only short version).

In the long version of the questionnaire, patient reported data on demographics, clinical characteristics, ATTR-FAP diagnostic experience, quality of life, functional impairment and ATTR-FAP management were collected. In the short version, the primary focus was on data collection related to quality of life and functional impairment. Both surveys are available online at https://medical-data-models.org/41269 (long version) and https://medical-data-models.org/41268 (short version).

### Data analysis

Global data was analyzed using Microsoft Excel for Mac (Version 16.25) and GraphPad Prism Version 8.21 (GraphPad Software, La Jolla, CA). Survey responses were summarized using descriptive statistics, including means, medians and percentages. In some instances, as indicated in the figure legends, responses are presented as scores compiled of individual responses.

## Data Availability

Surveys are available online at https://medical-data-models.org/41269 (long version) and https://medical-data-models.org/41268 (short version). The datasets used and analyzed during the current study are available from the corresponding author on reasonable request.

## Abbreviations

ATTR-FAP: Transthyretin familial amyloid polyneuropathy
TTR: Transthyretin
SD: Standard deviation
IQR: Interquartile range
